# Protective Effect of Probiotics against *Pseudomonas aeruginosa* Infection of Human Corneal Epithelial Cells

**DOI:** 10.3390/ijms25031770

**Published:** 2024-02-01

**Authors:** Irene Paterniti, Sarah Adriana Scuderi, Lucia Cambria, Antonia Nostro, Emanuela Esposito, Andreana Marino

**Affiliations:** Department of Chemical, Biological, Pharmaceutical and Environmental Sciences, University of Messina, 98166 Messina, Italy; ipaterniti@unime.it (I.P.); sascuderi@unime.it (S.A.S.); lucia.cambria1@studenti.unime.it (L.C.); antonia.nostro@unime.it (A.N.); eesposito@unime.it (E.E.)

**Keywords:** probiotic pretreatment, *Pseudomonas aeruginosa* infection, corneal epithelial cells, SkinEthic^TM^ HCE model

## Abstract

Probiotic therapy needs consideration as an alternative strategy to prevent and possibly treat corneal infection. This study aimed to assess the preventive effect of *Lactobacillus reuteri* and *Bifidobacterium longum* subsp. *infantis* on reducing the infection of human corneal epithelial (HCE) cells caused by *Pseudomonas aeruginosa*. The probiotics’ preventive effect against infection was evaluated in cell monolayers pretreated with each probiotic 1 h and 24 h prior to *P. aeruginosa* challenge followed by 1 h and 24 h of growth in combination. Cell adhesion, cytotoxicity, anti-inflammatory, and antinitrosative activities were evaluated. *L. reuteri* and *B. longum* adhered to HCE cells, preserved occludin tight junctions’ integrity, and increased mucin production on a SkinEthic^TM^ HCE model. Pretreatment with *L. reuteri* or *B. longum* significantly protected HCE cells from infection at 24 h, increasing cell viability at 110% (110.51 ± 5.15; *p* ≤ 0.05) and 137% (137.55 ± 11.97; *p* ≤ 0.05), respectively. Each probiotic showed anti-inflammatory and antinitrosative activities, reducing TNF-α level (*p* ≤ 0.001) and NO_x_ amount (*p* ≤ 0.001) and reestablishing IL-10 level (*p* ≤ 0.001). In conclusion, this study demonstrated that *L. reuteri* and *B. longum* exert protective effects in the context of corneal infection caused by *P. aeruginosa* by restoring cell viability and modulating inflammatory cytokine release.

## 1. Introduction

Infectious keratitis is considered a medical emergency as it can cause vision loss and blindness. Among the most common pathogens implicated in bacterial keratitis is *Pseudomonas aeruginosa*, which is of particular concern for several reasons [1,2]. It is highly virulent, difficult to treat, and results in worse corneal ulcers compared to those caused by other bacteria [3,4]. Predisposing factors for infectious keratitis include ocular disease, ocular injury, surgical procedures, and antibiotic therapy [5]. Moreover, *P. aeruginosa* infections can develop biofilms which facilitate a higher incidence of disease in extended-wear contact lens users [6,7]. Currently, treatment for several ocular surface infections is based on eye drops containing antibiotics that can stimulate changes in the healthy eye microbiota, contribute to the onset of eye diseases, and increase the resistance of pathogenic strains [8].

In healthy conditions, the ocular microbiota play an important role in maintaining local homeostasis: they modulate the immune response and, through a barrier effect, prevent the invasion and proliferation of pathogenic or opportunistic microorganisms [9,10]. The microbiome consists of bacteria, fungi, viruses, and protozoa. Recently, next-generation sequencing 16S rRNA technology has demonstrated the most abundant bacterial phyla are represented by *Proteobacteria* (64%), *Actinobacteria* (19.6%), and *Firmicutes* (3.9%) [10]. Most metagenomic sequencing results support *Corynebacterium*, *Propionibacterium*, and *Staphylococcus* as the dominant taxons of the healthy ocular surface. However, the microbiome and microbiota can undergo dynamic changes during the lifespan of a human being. The microbiota of the ocular surface can be affected by environmental conditions, age, sex, seasonality, personal habits, use of contact lenses, pathological states, infections, antibiotics, etc. [9].

Ocular surface dysbiosis, a substantial change in microbiota composition, can be associated to several diseases and conditions, including infections [2,11]. Keratitis caused by *P. aeruginosa* is refractory and difficult to treat because of its extensive and emerging antibiotic resistance. During the infection process, *P. aeruginosa* produces several virulence factors, forms a biofilm, and displays resistance to many antibiotics [12,13]. Biofilms are communities of microorganisms in nature that are attached to a biological or abiotic surface and are surrounded by a self-generated extracellular matrix that is mainly composed of polysaccharides, secreted proteins, and extracellular DNAs. Biofilm bacteria exhibit different phenotypic characteristics from their planktonic counterparts, including an increased resistance to antibiotics, and steep rises in the incidence of microbial keratitis have been linked to the increased popularity of contact lenses [13,14].

Treatment failure in clinical practice leads to the development of new therapeutic strategies. Among these strategies, probiotic therapy may have great potential in preventing and treating several ocular diseases [10,15]. According to a definition by the Food and Drug Administration (FDA), probiotics are classified as “live biotherapeutics: live microorganisms that have a positive impact on the health and physiology of the host” [16]. Numerous studies have proven that *Lactobacilli*, *Bifidobacteria*, *Escherichia coli*, *Saccharomyces cerevisiae*, *S. boulardii*, *S. lactis*, etc., could be used as probiotics [17,18]. Probiotics may modify immunological activity by increasing innate and adaptive immune responses, altering microbial habitats, producing antimicrobial compounds, and improving barrier function and competitive adhesion to the mucosa and epithelium [19]. Some strains can inhibit the adhesion of pathogens to the mucosa by forming a barrier via auto-aggregation or by direct coaggregation with the pathogens. Auto-aggregation facilitates the communication between cells as well as antagonism and estrangement of pathogenic bacteria [20]. Coaggregation allows for close interaction between the probiotic strain and the pathogen, and through the production of antimicrobial substances, the probiotic can inhibit the growth of pathogens [21,22].

Moreover, several probiotic strains form biofilms that can counteract the establishment of pathogenic biofilms [23].The main antimicrobial compounds produced by probiotic strains are organic acids, such as lactic, propionic, acetic, and succinic acid, and ethanol, hydrogen sulfide, biosurfactants, carbon dioxide, exopolysaccharides, and bacteriocins [24]. Probiotics are used to prevent and treat conditions such as diarrhea, acute pancreatitis, colon cancer, hypertension, diabetes, *Helicobacter pylori* infection, ventilator-associated pneumonia, migraine, and autism in clinical settings [19]. Nevertheless, in most cases, probiotic activity is strain-specific as demonstrated by Bubnov and colleagues [25] who investigated specific properties, such as adhesive ability and resistance to antibiotics, of lactic acid bacteria (LAB) and *Bifidobacteria*.

Nowadays, research on microbial biotherapy for ocular diseases is still in its infancy. However, some studies have focused their attention on how probiotics can restore the ocular microbiota and improve the course of some ocular surface diseases. Among these, some clinical trials reported the efficacy of topically applied probiotics such as *L. acidophilus* or *B. lactis* and *B. bifido* formulations to correct vernal keratoconjunctivitis or dry eye, respectively [26,27].

Currently, there are still few studies that investigate the effect of probiotics on ocular surface infections.

Probiotic therapy could promote a rapid and stable restoration of the healthy ocular microbiota, faster anti-inflammatory effect, and antagonistic action against pathogens [10].

The aim of this study was to evaluate the influence of *L. reuteri* and *B. longum* subsp. *infantis* on preventing *P. aeruginosa* infection of human corneal epithelial (HCE) cells. The experimental design involved two distinct phases in order to (i) determine the strain-specific characteristics of these probiotics, (ii) evaluate whether pretreatment of HCE cells with these probiotic strains can protect them from *P. aeruginosa* infection-induced damage.

## 2. Results

### 2.1. Strains and Growth Conditions

Overnight cultures of *L. reuteri* and *B. longum* strains in MRSB, with or without cysteine (0.05 g/L) and incubated at 37 °C under 5% CO_2_ conditions were diluted to obtain an optical density of 0.5 at 570 nm (equivalent to approximately 5 × 10^8^ CFU/mL). Both strains, with and without 0.05 g/L cysteine, showed the same concentrations when plated on MRSA (about 5 × 10^8^ CFU/mL). In the following assays, these strains were cultivated in MRSB or MRSA without cysteine at 37 °C under 5% CO_2_ conditions, in anticipation of subsequent tests where the strains would be put in contact with human corneal epithelial cells (HCE).

#### 2.1.1. Aggregation and Coaggregation with *P. aeruginosa*

**Figure 1:**
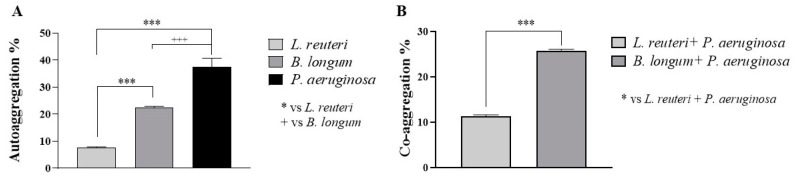
Percentage of auto-aggregation (**A**) and coaggregation with *P. aeruginosa* (**B**) of the probiotics after 5 h of contact. Results are expressed as mean ± SD (**A**) (*** *p* ≤ 0.001, +++ *p* ≤ 0.001); (**B**) (*** *p* ≤ 0.001).

The auto- and coaggregation abilities of the probiotics *L. reuteri* and *B. longum* elucidated in our study are summarized in Figure 1A,B. After 5 h of incubation, a significantly higher percentage of aggregation was observed for *B. longum* (22.35%) than for *L. reuteri* (7.70%). The best auto-aggregation ability was shown by *P. aeruginosa* (37.61%). *B. longum* also demonstrated a higher coaggregation ability with *P. aeruginosa* (25.59%) than *L. reuteri* (11.60%).

#### 2.1.2. Biofilm Production

**Figure 2:**
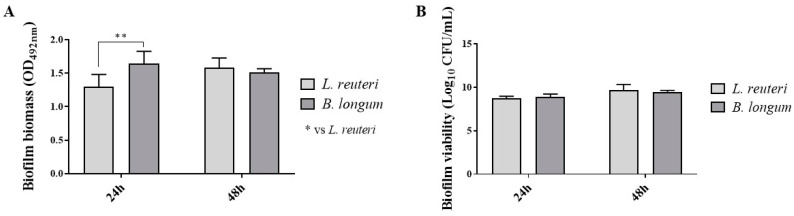
Biofilm formation ability of probiotics on polystyrene microplates. Percentage of biofilm biomass (**A**). Quantification of cell viability (**B**). Results are expressed as mean ± SD (**A**) (** *p* ≤ 0.01).

In this study, the probiotic strains’ ability to develop a biofilm was determined by evaluating biomass and viability in the sessile phase. As shown in Figure 2A, after 24 h of growth, *B. longum* showed a significantly greater ability to produce biomass with respect to *L. reuteri*. After 48 h of growth, *B. longum* and *L. reuteri* showed the same ability. In Figure 2B, the results of biofilm cell viability show that both strains reached the same colony-forming unit count after 24 h and 48 h of incubation.

#### 2.1.3. Effect on HCE Cell Viability

**Figure 3:**
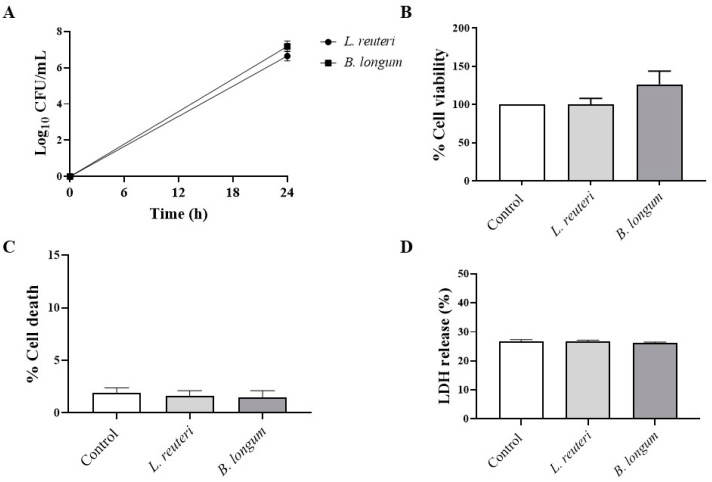
Growth curves of probiotics incubated with HCE cells at 24 h (**A**). Evaluation of effect of probiotics on HCE cell viability by MTT (**B**), trypan blue (**C**), and LDH (**D**) assays, respectively. Results are expressed as mean ± SD.

Before exploring the effects of each probiotic strain against *P. aeruginosa* infection, we assessed their effect on HCE cell viability via MTT assay. Figure 3A shows the growth curves of probiotics coincubated with HCE cells at 24 h. The coincubation of bacteria with HCE cells did not exert any cytotoxic effect (Figure 3B). The viability of HCE cells was 100% and 126% when *L. reuteri* and *B. longum* were added, respectively, compared to untreated HCE cells (Figure 3B). MTT results were confirmed by trypan blue staining (Figure 3C) and LDH assay (Figure 3D), demonstrating that *L. reuteri* and *B. longum* did not increase cell death, exerting a protective effect on HCE cells.

#### 2.1.4. Adhesion to HCE Cells

**Figure 4:**
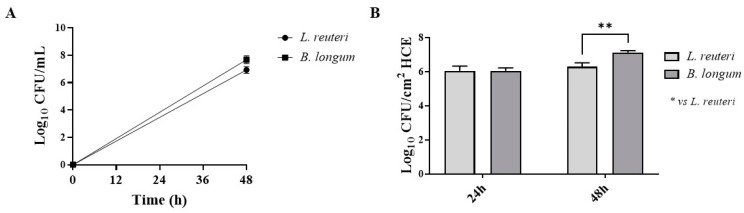
Growth curves of probiotics incubated with HCE at 48 h of contact (**A**). Quantification of probiotic bacteria adherent to HCE cells (**B**). Results are expressed as mean ± SD (**B**) (** *p* ≤ 0.01).

Probiotic strains with adhesion ability can prevent the adhesion of pathogens by competing for host cell binding sites [28]. The growth curves of probiotics incubated with HCE at 48 h of contact are shown in Figure 4A. As shown in Figure 4B, *L. reuteri* and *B. longum* demonstrated great ability to adhere to HCE cells after 24 h of contact, increasing the concentration of sessile cells after 48 h.

#### 2.1.5. Effect on Occludin and Mucin-1 in SkinEthic^TM^ HCE Model

**Figure 5:**
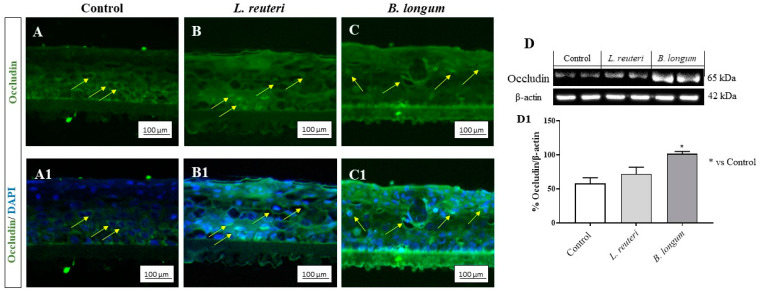
Evaluation of occludin staining on SkinEthic^TM^ HCE model. IF staining demonstrated that *L. reuteri* (**B**,**B1**) and *B. longum* (**C**,**C1**) guarantee the health of corneal structure compared to control group (**A**,**A1**). The results of IF were confirmed by Western blot analysis (**D**,**D1**). The yellow arrows indicate the positive staining. Results are expressed as mean ± SD (**D**,**D1**) (* *p* ≤ 0.05).

**Figure 6:**
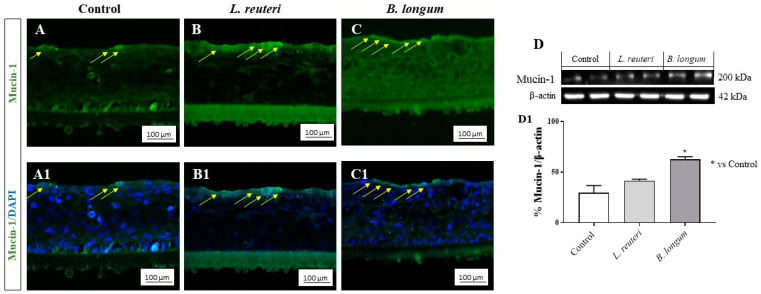
Evaluation of MUC-1 staining on SkinEthic^TM^ HCE model. IF staining demonstrated that *L. reuteri* (**B**,**B1**) and *B. longum* (**C**,**C1**) promote the health of corneal tissue compared to control group (**A**,**A1**). The results of IF were confirmed by Western blot analysis (**D**,**D1**). The yellow arrows indicate the positive staining. Results are expressed as mean ± SD (**D**,**D1**) (* *p* ≤ 0.05).

The cornea is characterized by the presence of intercellular junctions which contribute to the first line of protection against pathogens and allergens [29,30]. Thus, we decided to evaluate the effects of *L. reuteri* and *B. longum* on occludin level, a tight-junction-associated protein that is abundantly expressed in the corneal epithelium. As demonstrated in our study, the corneal epithelium of the control group showed basal levels of occludin staining, forming a continuous ring around the cells (Figure 5A,A1); the same condition was observed also in the *L. reuteri* and *B. longum* groups (Figure 5B,B1 and C,C1, respectively). Moreover, we investigated the effects of the probiotic strains on mucin-1 (MUC-1), a transmembrane glycoprotein expressed in the apical surface of corneal cells which plays multiple roles in the protection of mucosal surfaces [29]. The data showed that MUC-1 was abundantly expressed in the superficial layer of cornea in the control group (Figure 6A,A1) as well as in the *L. reuteri* and *B. longum* groups (Figure 6B,B1,C,C1), suggesting that these strains exert beneficial effects on corneal structure, promoting MUC-1 and occludin levels. The results of immunofluorescence (IF) for occludin and MUC-1 were confirmed by Western blot analysis, highlighting the beneficial effects of probiotics on the corneal epithelium as shown in Figure 5D and Figure 6D (see densitometric analysis Figure 5D1 and Figure 6D1, respectively).

### 2.2. Prevention of P. aeruginosa Infection Damage via Pretreatment of HCE Cells with Probiotic Strains

#### 2.2.1. Effect on HCE Cell Viability

**Figure 7:**
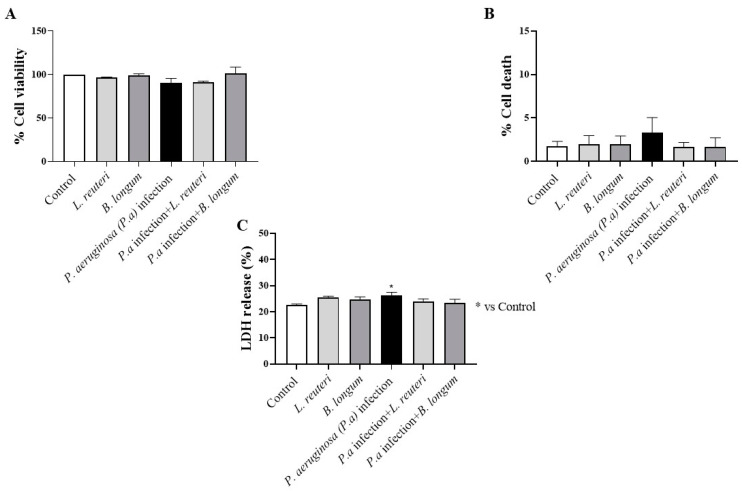
Effects of probiotics on HCE cell viability after *P. aeruginosa* infection at 1 h. *P. aeruginosa* infection did not exert any cytotoxicity on HCE cells compared to control and HCE cells treated with each probiotic alone at 1 h (**A**). Trypan blue staining (**B**) and LDH assay (**C**) were used to confirm MTT data. Results are expressed as mean ± SD (**C**) (* *p* ≤ 0.05).

HCE cells were treated with each probiotic strain for 1 h (short contact time) prior to *P. aeruginosa* inoculation. As shown in Figure 7A, *P. aeruginosa* infection (group 3) did not significantly reduce HCE cell viability compared to untreated HCE cells and HCE cells treated with each probiotic alone. MTT assay data were confirmed by trypan blue staining (Figure 7B) and LDH assay (Figure 7C), demonstrating that *P. aeruginosa* infection did not induce significant cell death or a marked LDH release after a short contact time.

**Figure 8:**
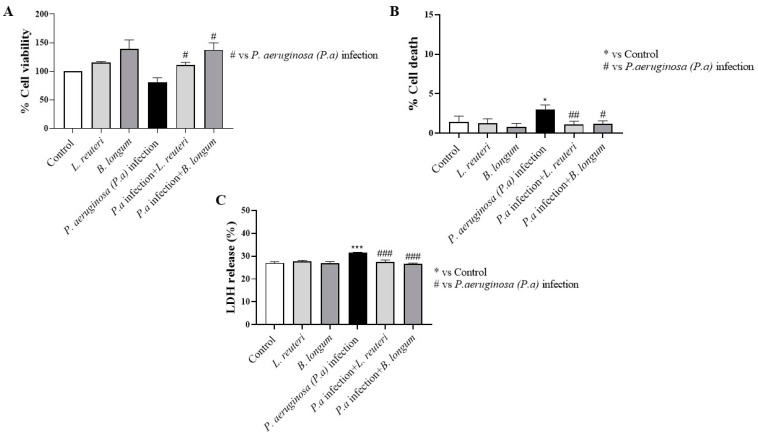
Effects of probiotics on HCE cell viability after *P. aeruginosa* infection at 24 h. Probiotics significantly restored HCE viability after *P. aeruginosa* infection at 24 h (**A**). Trypan blue staining (**B**) and LDH assay (**C**) were used to confirm MTT data. Results are expressed as mean ± SD (**A**) (# *p* ≤ 0.05); (**B**) (* *p* ≤ 0.05; # *p* ≤ 0.05; ## *p* ≤ 0.01); (**C**) (*** *p* ≤ 0.001; ### *p* ≤ 0.001).

The effect of probiotic strains on HCE cell viability was assessed at 24 h (long contact time) (Figure 8). The results showed that pretreatment with *L. reuteri* and *B. longum* (groups 3 and 4) significantly increased the viability of HCE cells compared to *P. aeruginosa* infection (group 3) (Figure 8A). MTT assay data were confirmed by trypan blue staining (Figure 8B) and LDH assay (Figure 8C), demonstrating that probiotic strains may reduce the % of cell death and LDH release compared to *P. aeruginosa* infection (group 3), exerting a protective effect.

#### 2.2.2. Antagonistic Activity against *P. aeruginosa* Adhesion

The results demonstrated that the probiotic strains did not prevent the adhesion of *P. aeruginosa* to HCE cells in the experimental conditions used in this study (Table 1).

#### 2.2.3. Anti-Inflammatory Activity against *P. aeruginosa*

**Figure 9:**
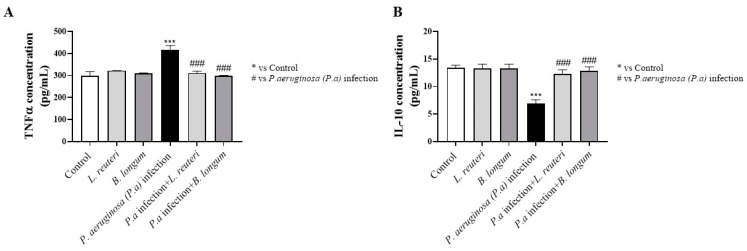
Evaluation of effect of probiotics on TNF-α and IL-10 levels. *P. aeruginosa* infection induced an increase in TNF-α level and a decrease in IL-10 level compared to control and to HCE cells treated with each probiotic alone. The probiotics significantly reduced TNF-α and restored IL-10 levels after *P. aeruginosa* infection at 24 h (**A**,**B**). Results are expressed as mean ± SD (**A**) (### *p* ≤ 0.001, *** *p* ≤ 0.001); (**B**) (*** *p* ≤ 0.001, ### *p* ≤ 0.001).

Considering the key role of the inflammatory process in corneal epithelial infections induced by *P. aeruginosa* [31], it was decided to investigate the effect of *L. reuteri* and *B. longum* at 24 h of coincubation on the levels of pro-inflammatory cytokines, such as tumor necrosis factor-α (TNF-α), and on levels of anti-inflammatory cytokines, such as interleukin (IL-10), in HCE cell supernatants. Our results demonstrated that *P. aeruginosa* infection (group 3) is characterized by an increase in the levels of the pro-inflammatory cytokine TNF-α and a decrease in the levels of the anti-inflammatory cytokine IL-10 compared to untreated HCE cells (group 1) and to HCE cells treated with each probiotic alone (group 2) (long contact time; see Figure 9A,B). However, *L. reuteri* and *B. longum* significantly reduced TNF-α and increased IL-10 levels compared to *P. aeruginosa* infection (group 3), as shown in Figure 9A,B, counteracting the inflammatory process.

#### 2.2.4. Antinitrosative Activity against *P. aeruginosa*

**Figure 10:**
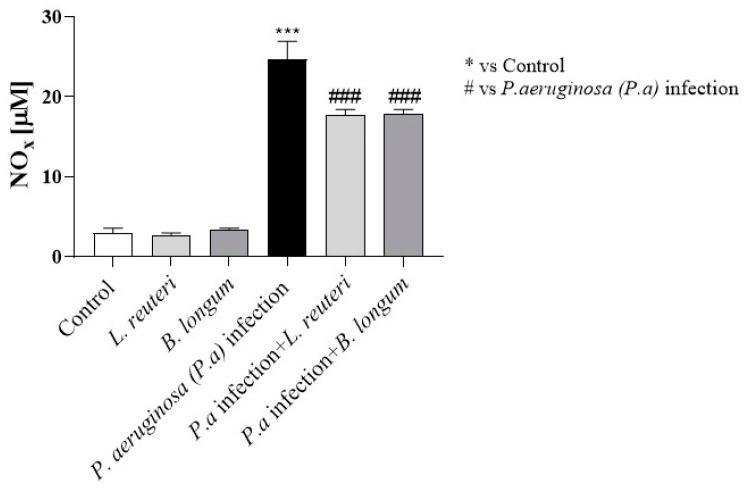
Effects of probiotics on NO_x_ levels. *P. aeruginosa* infection induced a significant increase in NO_x_ levels compared to control and HCE cells treated with each probiotic alone at 24 h. The probiotics significantly reduced NO_x_ levels at 24 h of coincubation. Results are expressed as mean ± SD (*** *p* ≤ 0.001, ### *p* ≤ 0.001).

Nitrosative stress is a process characterized by the overproduction of nitric oxide (NO); it is characterized by the simultaneous production of superoxide anions, which results in the formation of peroxynitrite, and of other reactive nitrogen species (RNS), which contribute to mitochondrial dysfunction and inflammatory process [32,33,34]. Based on these findings, the effect of probiotics on NO_x_ levels in HCE cell supernatants following *P. aeruginosa* infection was investigated. HCE cells were treated with each probiotic strain for 24 h (long contact time) prior to *P. aeruginosa* inoculation. At 24 h of coincubation, the supernatants were collected and analyzed. As shown in Figure 10, the results revealed that *P. aeruginosa* infection (group 3) was characterized by a significant increase in NO_x_ levels compared to untreated HCE cells (group 1) and HCE cells treated with each probiotic alone (group 2); however, *L. reuteri* and *B. longum* significantly reduced NO_x_ levels (group 4) at 24 h (long contact time; see Figure 10), counteracting nitrosative stress.

## 3. Discussion

Keratitis caused by *P. aeruginosa* is of particular concern because it develops rapidly, triggering an inflammatory/immune reaction that may lead to vision loss after ocular trauma or in contact lens wearers [35]. Traditional strategies for infection include antibiotics and steroids in the form of eye drops. However, eye drops containing antibiotics may lead to dysbiosis, causing an imbalance in favor of pathogenic strains and increasing the risk of resistance [10,36,37]. Those containing steroids may bring side effects, such as increased intraocular pressure or corneal complications. Probiotics could be one of the most promising biotherapies in the prophylaxis of and therapeutic usage for a variety of diseases, including infections [10]. The goal of prophylactic probiotics is to keep “good” bacteria colonyzed to combat pathogen invasion [19]. Several experimental and clinical results have shown the benefits of probiotics in the prevention of *P. aeruginosa* infection [38,39,40]. Preventing overt microbiological damage or imbalance may determine improvement in clinical illness as well as reduce the frequency and prevalence of the chronic infections [41].

In line with this, our results demonstrated that pretreatment with probiotic strains significantly protected HCE cells by reducing damage caused by *P. aeruginosa* infection. We demonstrated that probiotic strains preserved HCE cell viability, promoting occludin distribution and mucin production, suggesting their beneficial effects on HCE tissue structure. Regarding the efficacy of pretreating HCE cells with probiotic strains before *P. aeruginosa* infection, a significant restoration of cell viability was demonstrated compared to untreated and infected HCE cells. Moreover, *L. reuteri* and *B. longum* significantly reduced the levels of pro-inflammatory cytokines such as TNF-α and reestablished IL-10 levels, reducing the inflammatory response following a long contact time. In addition, with reference to antinitrosative activity, pretreatment with the probiotic strains showed a significant reduction in NO_x_ level, counteracting the overproduction of nitrogen-based free radicals at 24 h of pretreatment. However, the adhesion of *P. aeruginosa* to HCE was not countered by probiotic strains, probably because of their modest capacity for auto-aggregation, coaggregation and adhesion with *P. aeruginosa*.

Many studies have proven the beneficial effects of probiotics on many diseases, achieved by promoting cell survival, cell proliferation, barrier function, and by stimulating the immune response [42,43,44,45,46]. Pretreatment with probiotic bacteria such as *Lactobacilli* exerts antioxidant and antiapoptotic activity, both in vitro and in vivo, improving cell viability [47,48]. Recent reports demonstrated that the application of probiotics might be effective in the management of eye diseases, suggesting probiotics as a potential alternative therapeutic treatment thanks to their ability to regulate epithelial barriers, secrete mucus and antimicrobial peptides, as well as to stimulate cell proliferation and the immune system [49].

Among the strains commonly found in the human microbiota, lactobacilli are mainly predominant in the gastric region and upper gastro-intestinal tract, while bifidobacteria in the lower intestinal tract. *L. reuteri,* used in this study, belongs to clade II. It has been demonstrated that clade II strains can be considered immunosuppressive and anti-inflammatory [50,51,52]. Moreover, *L. reuteri* can attach to mucin and suppress potentially deleterious effects of mucosal inflammation but it produces low levels of antimicrobial compounds such as reuterin [53,54,55,56]. *B. longum* subsp. *infantis*, within the genus *Bifidobacterium,* has shown several beneficial effects attributed to strains belonging to the subspecies [57]. This strain has proven to be particularly effective in protecting against infectious diseases and in increasing the maturation of the immune response to suppress inflammation [58,59,60]. In light of this, a recent clinical study demonstrated that the application of *Lactobacillus* and *Bifidobacterium* strains could be a potential alternative therapeutic treatment for eye diseases by modulating the immune system, cell proliferation, and the secretion of mucus [49].

In this study, it was demonstrated that topical pretreatment of HCE cells infected with *P. aeruginosa* with *L. reuteri* or *B. longum* subsp. *infantis* can restore cell viability and reduce the inflammatory reaction caused by the pathogen, while also showing an antinitrosative effect. The prophylactic topical administration of a probiotic or a mixture of probiotic strains can reduce the number, duration, and severity of recurrent keratitis caused by *P. aeruginosa* and prevent ulcer formation. However, further studies are necessary to clarify the mechanisms by which probiotic strains act in preventing *P. aeruginosa* infection.

Considering the obtained in vitro data, in the next study, we will evaluate these benefit activities in an in vivo model. At this moment, in the field of utilizing probiotics as a new therapy for eye infection, much remains to be done. More experimental studies are needed to investigate the mode of action of homeostatic microorganisms in the prevention of several ophthalmic diseases. Knowing the efficacy and safety of single probiotic strains in contact with HCE cells could lead to the formulation of a mixture of probiotic strains with different properties that could synergistically assist antibiotic and/or anti-inflammatory drugs in preventing or treating corneal infections.

## 4. Materials and Methods

### 4.1. Strains and Growth Conditions

The probiotic strains tested in this study were *L. reuteri* DSM20016 and *B. longum* subsp. *infantis* DSM20088 (Leibniz Institute DSMZ-German Collection of Microorganisms and Cell Cultures, Braunschweig, Germany) [53,61]. The pathogen strain used was *P. aeruginosa* American Type Culture Collections (ATCC 9027). The *L. reuteri* and *B. longum* strains were grown in De Man, Rogosa, and Sharpe broth (MRSB, Oxoid, Milan, Italy) with and without the addition of 0.05% cysteine for 24–48 h at 37 °C under 5% CO_2_ conditions. *P. aeruginosa* was grown in tryptic soy broth (TSB, Oxoid) at 37 °C for 24 h in aerobic conditions. The strains were stored at −70 °C in Microbanks™ (Pro-lab Diagnostics, Neston, UK). All reagents were purchased from Sigma-Aldrich (Milan, Italy) unless otherwise specified in the text.

### 4.2. Aggregation and Coaggregation with P. aeruginosa

Auto-aggregation assay. The cultures of probiotic strains in steady state were centrifugated at 10,000× *g* for 15 min and the bacteria were resuspended in 10 mL of phosphate-buffered saline solution (PBS) (pH 7.4) to approximately 2 × 10^8^ CFU/mL (OD at 600 nm 0.5 for *L. reuteri* and *B. longum* strains). Each suspension was vortexed for 10 s and incubated for 5 h at room temperature. At each hour, 1 mL of the upper part of each suspension was withdrawn to measure absorbance. The percentage of auto-aggregation was calculated according to the following formula:

Auto-aggregation% = [1 − OD_t_/OD_i_] × 100

where OD_i_ is the initial OD of the microbial suspension and OD_t_ is optical density at different time points.

Coaggregation assay. The suspensions of probiotic strains were treated as mentioned above. *P. aeruginosa* was harvested in stationary phase by centrifugation during 10 min at 5000× *g* and resuspended in PBS (pH 7.2) to obtain a *P. aeruginosa* suspension of about 2 × 10^8^ CFU/mL (OD_600_ 0.2). Aliquots of pairs of bacterial suspensions (each probiotic and pathogen strain) totaling 10 mL each were vortexed for 10 s. Samples containing a single bacterial suspension were used as control. Each suspension was vortexed for 10 s and maintained for 5 h at room temperature. At each hour, 1 mL of the upper part of each suspension was withdrawn to measure absorbance as described above. The percentage of coaggregation was then calculated according to the following formula:

$$\mathrm{Co-aggregation}\%=\frac{[(\mathrm{Ax}+\mathrm{Ay})/2]-\mathrm{At}(x+y)\times 100}{(\mathrm{Ax}+\mathrm{Ay})/2}$$

where Ax (pathogen) and Ay (probiotic) are the OD values of each probiotic or pathogen strain suspension, and At (x + y) is the OD of the combined aggregation of each probiotic strain with the pathogen at different time points [62].

### 4.3. Biofilm Production

The evaluation of biofilm formation was carried out by determining the biomass and viability of each probiotic strain in the sessile phase. Overnight cultures of *L. reuteri* and *B. longum* strains in both MRSB and MRSB with 0.05% cysteine were standardized to a concentration of 5 × 10^5^ CFU/mL, placed in a flat-bottomed polystyrene microplate (Corning Inc., Corning, NY, USA), and incubated for 24–48 h at 37 °C under 5% CO_2_ conditions. Then, the planktonic phase was gently removed, and the wells were washed with PBS (pH 7.4) 3 times. To assess the biomass of the strains, the microplate wells were dried, colored with 0.1% safranin, and washed again with PBS. The colored biofilm was resuspended in 30% acetic acid (*v*/*v*) and then OD_492_ was measured by spectrophotometer. Control wells containing only the medium were included. The production of biofilm was obtained by comparing the OD value obtained from the arithmetic mean of the values of each strain with the cut-off value (ODc), defined as the average of the values that was obtained from the control. To assess the cell viability of the samples, microplate wells with adherent biofilm were scarified in PBS, serially diluted, and seeded on MRSA (Oxoid) plates. After an incubation period of 24 h and 48 h at 37 °C, CFU/mL were counted [63,64].

### 4.4. Cell Culture

Human corneal epithelial (HCE) cells were kindly provided by the Sooft Research Center, Catania, Italy, SpA. HCE cells were isolated from the human cornea of a donor patient as described by Cristaldi and colleagues [65]. HCE cells were cultured in 75 cm^2^ flasks in DMEM-F12 Advanced (ATCC, cat. no. 12634010) and supplemented with 1% penicillin/streptomycin (penicillin 1000 units–streptomycin 0.1 mg/L, Sigma-Aldrich^®^ Catalog No. P4333; St. Louis, MO, USA), 2% fetal bovine serum (FBS) (Sigma-Aldrich, St. Louis, MO, USA, cat. no. F7524), and specific corneal epithelial growth factors (ATCC, cat. no. PCS-700-040) at 37 °C in a humidified atmosphere containing 5% CO_2_.

### 4.5. Viability Assays

Cell viability was measured using a mitochondria-dependent dye for live cells ((3-(4,5 Dimethylthiazol-2-yl)-2,5-diphenyltetrazolium bromide); MTT), lactate dehydrogenase (LDH) assay, and trypan blue staining. For MTT assay, HCE cells were seeded at a density of 4 × 10^4^ cells/well in 96-well plates for each probiotic or pathogen in DMEM without antibiotic supplements and incubated at 37 °C and 5% CO_2_ in the incubator. After 24 h, the cells were incubated with each probiotic strain suspension (1 × 10^7^ CFU/mL) [66,67]. After 24 h, HCE cells were incubated at 37 °C with MTT (0.2 mg/mL) for 1 h. The medium was removed by aspiration and the cells were lysed with DMSO (100 μL). Absorbance (OD_570_) was measured using a microplate reader [68].

To confirm MTT results, cell membrane integrity was analyzed using the cytotoxicity assay (G1780, Promega, Madison, WI, USA) according to the manufacturer’s instructions. The percentage of LDH released was calculated using the following formula:

$$\%\mathrm{LDH}\;\mathrm{release}=\frac{100\times \mathrm{Experimental}\;\mathrm{LDH}\;\mathrm{release}\;\left(OD_{490}\right)}{(\mathrm{Maximum}\;\mathrm{LDH}\;\mathrm{release}\;\left(OD_{490}\right)}$$

For trypan blue staining, after incubation with probiotic strains, trypan blue solution (0.4%) was mixed with trypsinized cell suspensions at a ratio of 1:1 to evaluate the % of cell death. After 5 min incubation, cells were loaded onto a hemocytometer, and both live (unstained) and dead (blue-stained) cells were counted under a light microscope [69]. The number of cells died was expressed as %.

### 4.6. Adhesion to HCE Cells

Bacterial adhesion tests on HCE cell monolayers were carried out in 96-well tissue culture plates. Cells were grown routinely in DMEM, supplemented as mentioned above without antibiotic to reach a density of 4 × 10^4^ cells/well in 96-well plates, and incubated at 37 °C under 5% CO_2_ conditions for 24 h until the formation of a dense cell layer. Probiotic strains were cultivated for 24 h in a standard culture broth before the adhesion experiment. The bacterial suspension was then washed twice in PBS and finally resuspended in DMEM to a 1 × 10^7^ CFU/mL final concentration. Subsequently, each bacterial suspension was added to the wells of the microtiter in monolayers and incubated at 37 °C under 5% CO_2_ conditions for 24 and 48 h. The supernatant of each probiotic strain was eliminated and unattached cells were gently removed by PBS buffer 3 times. The cells were then lysed by 0.1% Triton X-100 lysis buffer (10 mM Tris-HCl, 2 mM MgCl_2_, 0.25% Triton X-100, pH 8.0) and the number of viable adherent bacteria was determined by plating serial dilutions on MRSA to quantify the total number of cell-associated bacteria (CFU/cm^2^) [67,70].

### 4.7. SkinEthic^TM^ HCE Model

The SkinEthic^TM^ HCE model was purchased from EPISKIN Laboratories (Lyon, France, UE). It is an in vitro model of transformed human corneal keratinocytes cultivated on an inert polycarbonate filter which is structurally, morphologically, and functionally like the human cornea, with the presence of basal and wing cells and mucus production [71]. The SkinEthic^TM^ HCE model was cultured in a chemically defined medium as previously described [71].

Experimental groups:

- SkinEthic^TM^ HCE cells in DMEM (control group);
- SkinEthic^TM^ HCE cells treated with each probiotic strain at 1 × 10^7^ CFU/mL for 24 h at 37 °C, under 5% CO_2_ conditions.

### 4.8. Immunofluorescence (IF) Staining for Intercellular Junctions and Mucin Localization

SkinEthic^TM^ HCE sections of 7 μm were incubated with the primary antibodies antioccludin (1:100; 71-1500 Invitrogen, Carlsbad, CA, USA) or antimucin 1 (1:200; sc-7313, Santa Cruz Biotechnology, Santa Cruz, CA, USA) in a humidified chamber at 37 °C overnight. Sections were washed three times with PBS and were incubated with secondary antibody Alexa Fluor-488 (1:1000 in PBS, *v*/*v*, Molecular Probes, Altrincham, UK) for 1 h at 37 °C. Sections were washed in PBS and, for nuclear staining, 4′,6′-diamidino-2-phenylindole (DAPI; Hoechst, Frankfurt, Germany) (2 μg/mL) was added to PBS [72]. Sections were observed and photographed at 100× magnification using a Leica DM2000 microscope (Wetzlar, Germany).

### 4.9. Western Blot Analysis

Western blot analysis was performed on SkinEthic^TM^ HCE cells as previously described [73]. Protein concentration was estimated by the Bio-Rad protein assay using bovine serum albumin (BSA) as standard. Samples were heated at 95 °C for 5 min, and the same amounts of protein were separated on 12% SDS-PAGE gel and blotted to PVDF membrane (Immobilon-P). Membranes were incubated overnight (O/N) at 4 °C with the following primary antibodies: anti-occludin (1:500, Cat#91131, Cell signaling, Danvers, MA, USA) or anti-mucin 1 (1:500; sc-7313, Santa Cruz Biotechnology, Santa Cruz, CA, USA). To ascertain that the blots were loaded with equal amounts of proteins, the membranes were also incubated in the presence of the antibody against β-actin protein (1:1000; sc-47778, Santa Cruz Biotechnology, Santa Cruz, CA, USA). Results are expressed as % of the control.

### 4.10. Pretreatment of HCE Cells with Probiotic Strains Prevents P. aeruginosa Infection Damage

After washing the cell monolayers 3 times with PBS, each probiotic strain (approximately 1 × 10^7^ CFU/mL) was added 1 h (short contact time) and 24 h (long contact time) prior to *P. aeruginosa* inoculation (1 × 10^6^ CFU/mL). Then, the short-contact-time group was incubated for 1 h and the long-contact-time group for 24 h at 37 °C under 5% CO_2_ conditions. Control wells (HCE cells in medium) and HCE cells with *P. aeruginosa* challenge were included.

Experimental groups:

1. HCE cells in DMEM (control group);
2. HCE cells pretreated with each probiotic strain (pretreated cells);
3. HCE cells infected with *P. aeruginosa* (infected cells);
4. HCE cells pretreated with each probiotic strain and then infected with *P. aeruginosa* (pretreated and infected cells).

### 4.11. Viability Assays

After 1 h (short contact time) and 24 h (long contact time) of growth, the HCE control monolayer (group 1), pretreated monolayers (group 2), monolayers infected with *P. aeruginosa* (group 3), and pretreated monolayers infected with *P. aeruginosa* (group 4) were incubated at 37 °C with MTT. To confirm MTT results, LDH assay and trypan blue staining were performed following the above-described protocol.

### 4.12. Antagonistic Activity against P. aeruginosa Adhesion to HCE Cells

After 1 h (short contact time) and 24 h (long contact time) of growth, the HCE control monolayer (group 1), pretreated monolayers (group 2), monolayers infected with *P. aeruginosa* (group 3), and pretreated monolayers infected with *P. aeruginosa* (group 4) were washed twice with PBS and lysed with 1 mL/L (*v*/*v*) Triton X-100 for 5 min and were serially diluted. Bacteria were determined by plate counting on MRSA for *L. reuteri* and *B. longum* strains and on *Pseudomonas* agar base (Oxoid) for *P. aeruginosa*. The total number of cell-associated bacteria was expressed as CFU/cm^2^ [74,75].

### 4.13. Enzyme-Linked Immunosorbent Assay for TNFα and IL-10 in HCE Cell Supernatant

At 24 h (long contact time), the anti-inflammatory effect on pretreated HCE monolayers infected with *P. aeruginosa* (group 4), monolayers infected with *P. aeruginosa* (group 3), pretreated monolayers (group 2), and the control monolayer (group 1) were evaluated by enzyme-linked immunosorbent assay (ELISA). ELISA kits for tumor necrosis factor-α (TNF-α) and interleukin-10 (IL-10) were used according to the manufacturer’s instructions (TNF-α ELISA Kit My BioSource; cat No. MBS267654; IL-10 ELISA Kit eBioscience; Catalog Number: 88-7106). Cell supernatants were centrifuged for 10 min at 3000 rpm; then, supernatants were collected for immediate testing and/or stored at −20 °C. Absorbance was measured at 450 nm. The authors decided to evaluate cytokines release only at 24 h (long contact time) due to cell viability results.

### 4.14. Measurement of NO_x_ Levels in HCE Cell Supernatant

At 24 h (long contact time), total nitrite levels, as an indicator of nitric oxide (NO) production, were measured in pretreated HCE monolayers infected with *P. aeruginosa* (group 4), monolayers infected with *P. aeruginosa* (group 3), pretreated monolayers (group 2), and the control monolayer (group 1) in the supernatant as previously described [76]. Briefly, nitrate in the medium was reduced to nitrite by incubation with nitrate reductase (670 mU/mL) and β-nicotinamide adenine dinucleotide 3-phosphate (160 mM) at room temperature for 3 h. Entire nitrite concentration was later calculated via the Griess reaction by adding 100 μL Griess reagent [0.1% (*w*/*v*) N-(1-naphthyl) ethylenediamine dihydrochloride in H_2_O and 1% (*w*/*v)* sulfanilamide in 5% (*v*/*v*) concentrated H_3_PO_4_; volume 1:1] to the 100-μL sample. OD_570_ was determined using a microplate reader (Tecan, Männedorf, Switzerland). The authors decided to evaluate NO_x_ levels only at 24 h (long contact time) due to cell viability results.

### 4.15. Statistical Analysis

The results from the 3 experiments, expressed as means ± standard deviation, were statistically analyzed using the software GraphPad Prism 9.5.1. The results were analyzed using a one-way analysis of variance (ANOVA) and *t*-test procedure. Multiple comparisons were performed among groups by the Bonferroni correction test. Differences were considered statistically significant at *p* ≤ 0.05.

## 5. Conclusions

In conclusion, based on the obtained results, we demonstrated that *L. reuteri* and *B. longum* exert several beneficial effects in the context of corneal infection caused by *P. aeruginosa* thanks to their ability to restore cell viability and modulate inflammatory and nitrosative processes. However, despite these interesting data, further studies are needed to better understand their efficacy and mechanisms in the process of corneal infection.

## Figures and Tables

**Table 1:** Adhesion of *P. aeruginosa* to HCE cells in association with each probiotic strain with respect to controls.

Contact Time	*P.a*.	*P.a. + L.r.*	*P.a.* + *B.l*
Short	7.1 ± 0.2	6.4 ± 0.6	6.5 ± 0.1
Long	8.8 ± 0.4	8.7 ± 0.3	8.4 ± 0.5

*P.a.* = *Pseudomonas aeruginosa*; *L.r.* = *Lactobacillus reuteri*; *B.l.* = *Bifidobacterium longum*. Results (expressed in units of Log_10_ CFU/cm^2^) are expressed as mean ± SD.

## Data Availability

All data generated or analyzed during this study are included in this article.
